# Predicting *In Silico* Which Mixtures of the Natural Products of Plants Might Most Effectively Kill Human Leukemia Cells?

**DOI:** 10.1155/2013/801501

**Published:** 2013-01-28

**Authors:** Hany A. El-Shemy, Khalid M. Aboul-Enein, David A. Lightfoot

**Affiliations:** ^1^Faculty of Agriculture Research Park (FARP) and Department of Biochemistry, Faculty of Agriculture, Cairo University, Giza 12513, Egypt; ^2^Department of Clinical Pathology, National Cancer Institute, Cairo University, Giza 12513, Egypt; ^3^Genomics Core Facility, Department of Plant Soil and Agricultural Systems, SIUC, Carbondale, IL 62901, USA

## Abstract

The aim of the analysis of just 13 natural products of plants was to predict the most likely effective artificial mixtures of 2-3 most effective natural products on leukemia cells from over 364 possible mixtures. The natural product selected included resveratrol, honokiol, chrysin, limonene, cholecalciferol, cerulenin, aloe emodin, and salicin and had over 600 potential protein targets. Target profiling used the Ontomine set of tools for literature searches of potential binding proteins, binding constant predictions, binding site predictions, and pathway network pattern analysis. The analyses indicated that 6 of the 13 natural products predicted binding proteins which were important targets for established cancer treatments. Improvements in effectiveness were predicted for artificial combinations of 2 or 3 natural products. That effect might be attributed to drug synergism rather than increased numbers of binding proteins bound (dose effects). Among natural products, the combinations of aloe emodin with mevinolin and honokiol were predicted to be the most effective combination for AML-related predicted binding proteins. Therefore, plant extracts may in future provide more effective medicines than the single purified natural products of modern medicine, in some cases.

## 1. Introduction

Plant-derived secondary metabolites have been used to treat acute infections, health disorders, and chronic illness for tens of thousands of years. Only during the last 100 years have natural products been largely replaced by synthetic drugs [1]. However, important anticancer agents have to be extracted from plants, due to their complex structures that often contain several chiral centers. Further, some patients show resistance to known treatments [2]. Therefore, new treatments with different modes of action are constantly sought. Plants are an abundant source of new natural products. Estimates of 200,000 natural products in plant species have been revised upward as mass spectrometry techniques have developed [3]. New databases, omics methods, and good practice standards are promising to deliver many new medicines based on plant natural products [4].

Several studies have demonstrated that mixtures in extracts from herbal medicines had anticancer potential *in vitro* or *in vivo *[5–7]. Among many other studies, aqueous extracts from willow (*salix* sp.; Salicaeae) leaves prevented proliferation of three cancer cell types acute myeloid leukemia (AML), acute lymphoblastic leukemia (ALL), and Ehrlich ascites carcinoma cells [7]. Therefore, the complex mixtures in crude extracts may be more effective than single purified natural products.

 Leukemia was among the most common cancers throughout human history [8]. However, the greater prevalence of leukemia in the modern world may be due to the reduction of infectious diseases that lead to the increased life span for most human populations. Unfortunately, by 2012 treatments for cancer diseases were expensive with no assurance that even simple leukemia can be cured. 

For developing countries the identification and use of endogenous medicinal plantsas cures against leukemia and other cancers have become attractive [9]. In developed countries the use of *in silico* analysis to predict useful new treatments and potential side-effects has risen to prominence [4, 5, 10]. Here, the two approaches were combined. 

Single natural products with well-known bioactivities [11, 12] were used in this study. The objectives here were to identify potential antileukemic compounds; to predict effective combinations of natural products; and to rank them on the basis of putative antileukemic activity. In addition the *in silico* analysis sought to identify target protein(s) for potentially antileukemic natural products; to predict the modes of action of those compounds; to predict potential adverse drug reactions (toxicity); and to predict the absorption, distribution, metabolism, and excretion (ADME) profiles. 

## 2. Materials and Methods

### 2.1. Databases and Software

**Figure 1:**
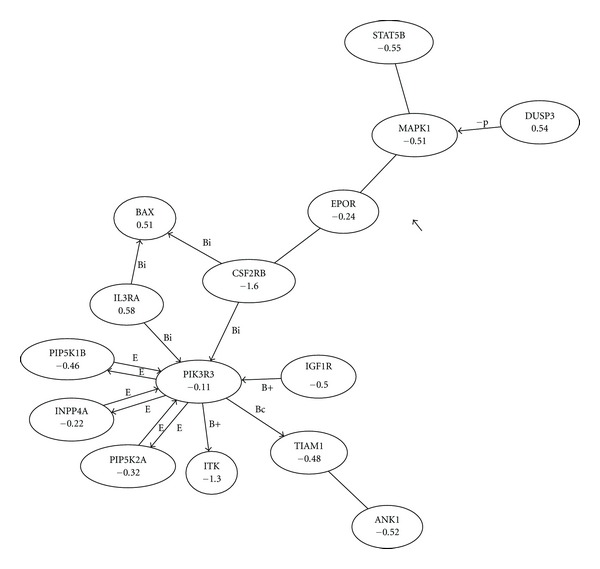
Subnetwork dysregulated in AML versus normal white blood cells. Interaction-type annotations from KEGG were shown as the letters above the arrows where; E was enzymatic; T was transcription with subscript + showing activation and − showing inhibition; B was protein-to-protein binding. Subscripts for the predicted protein-to-protein interactions were c: for compound interactions, +: activation, −: inhibition, i: an indirect effect, s: a state change, p+: phosphorylation, p−: dephosphorylation, m: methylation, u: ubiquitination, g: glycosylation and “none” for missing information.

The reference databases and software of Ontomine were used for predictive analysis. Ontomine was chosen because it provided an innovative chemoinformatics prediction tool based on the presence or absence of chemical group(s) of a set of related natural products. Ontomine searches were performed against large and manually curated databases. They included (i) *Literature searches* based on experimentally determined properties from around 100.000 diverse small molecules, collected from databases, encyclopedias, and other literature followed by expert hand-curation; (ii) *BioAssay Knowledgebase* that was compiled from over 500 bioassay data found at NCBI-PubChem; (iii) *Target Protein Knowledgebase* that was compiled by curation among the ~1500 proteins from DrugBank at NCBI-PubChem (details given in Figure 1). (iv) *Pathway Analysis*; KEGG pathways were used as references (ftp://ftp.genome.jp/pub/kegg/); (v) *Docking Algorithms* were used to identify molecular binding sites and predict ligand binding constants. Ontomine databases and tools are among those used widely in this field [4].

### 2.2. Natural Products Selected for Prediction of the Basis of Antileukemia Activity

Thirteen commercially available, purified, natural products of plants were selected to be tested for their antileukemic (AML) properties (Table 1). Natural products had been shown to cause some cell death when incubated with a primary AML cell lines for 24 hrs at low concentrations (Supplemental Table  1 see Supplementary materials available online at http://dx.doi.org/10.1155/2013/801501). Mortality rate and dose dependence were known [7], but bioassays had not clearly identified the best therapeutics, and the *in vivo* analysis of all mixtures would have been costly. Therefore, *in silico* prediction was used to identify the best candidates for later testing *in vivo*. A parallel analysis was made with established drugs for AML treatments so that the predictions could be compared. 

### 2.3. Ontomine-Based Analyses of Functional Groups

 Ontomine was used to transform the structural information for chemically, biologically, or pharmacologically related molecules to a hierarchical schema of functional groups. It was used to discover patterns in the related schema and predict biological activity, toxicity, and side-effects using rules inferred from analyzing the patterns. The basic algorithms underlying Ontomine-based predictions were as follows. 

 For rule or pattern detection the cluster formation was based on a *similarity threshold (ST) that was *calculated for each molecule using the formula:

(1) $$\begin{array}{c} \frac{\text{No}.\;\text{of}\;\text{common}\;\text{functional}\;\text{groups}\;\text{in}\;\text{molecule}\;1\;\text{and}\;2}{\text{Maximum}\;\text{functional}\;\text{group}\;\text{count}\;\text{in}\;\text{either}\;\text{molecule}} \end{array}$$

Once ST was calculated, clusters formed if the ST was less than or equal to 0.7. Two clusters could be merged if each contained the same molecules or a subset thereof. Results were generated with confidence levels of high, medium, and low.

The Ontomine algorithm took into account presence and absence of functional groups among sets of related molecules (with similar bioactivity or toxicity) to derive rules for making predictions. This approach was different from traditional maximum common subgraphs (MCS) approaches in which only a part of molecule is taken into consideration for decision making. 

### 2.4. Drug Target Analysis

The database used contained bioassay data for ~500 predicted binding proteins (Supplemental Figure  1). Natural product target identification was done by mining two specific knowledgebases: the “Drug Target KB” and “BioAssay KB”. BioAssay KB was generated from 493 bioassay records from NCBI and Drug Target KB (1,346 records) was generated from PubChem, DrugBank, and TTD [4]. Predicted binding proteins for 13 natural products were predicted at three confidence levels of high, medium, and low. The predicted bioassays were validated using scientific journals, and some of the bioassay targets were found as active against AML in the literature [13–24].

### 2.5. Reverse Docking to Predict Binding Affinities for Proteins

 Docking was a computational method used to estimate binding strength among biomolecules (protein-ligand and protein-protein interactions). Traditionally docking was used as computational tool for screening databases of natural products to mine a set of a few candidate drug-like compounds. Reverse docking was a comparatively new application of docking in which a database of proteins (drug targets) was docked against a set of natural products (potentially anticancerous compound), to predict binding affinities. AutoDock4.0 software was used to carry out reverse docking over a database of ~1,100 predicted binding proteins available in the Potential Drug Target Database (PDTD) [10]. The PDTD contained more than 1,100 protein entries with known 3D structures. The PDTD covered diverse information of more than 830 known or potential drug targets, including protein and active site structures, related diseases, biological functions, and their associated regulating (signaling) pathways. Taking every ligand as a probe, reverse docking was carried out with the entire database (PDTD). The respective ligands were also prepared using AutoDock4.0. A grid of each protein's binding pocket (site) was constructed using the Autogrid module of AutoDock4.0. Every ligand was separately docked into the binding site of each protein. The interaction energies between the ligand and the proteins were calculated in the form of docking scores. AutoDock4.0 also yielded the inhibition constant (*K*_*i*_) for every docking calculation. The predicted binding proteins were screened using a cut off value of <10 *μ*M for the inhibition constant (*K*_*i*_).

### 2.6. Pathway Analyses

Pathway analyses were performed on predicted binding proteins, to provide insights into potential mechanisms of natural product activity. Three statistical confidence levels were considered for pathway analyses. Statistical analysis used Fishers Exact Test to identify overrepresented pathways. 

### 2.7. Gene Expression Network Analysis

 Protein-protein interaction network analyses can provide important information about disease mechanisms and help to select candidates for genes and their encoded proteins underlying disease. Networks can include transcript and protein co-expression data. Here AML was compared to normal white blood cell transcript abundance data from published studies [12, 25] available within the NCBI-GEO database (Gene Expression Omnibus) (Accession: GSE17054, GSE9476). The algorithm described [25] for finding significant subnetwork/modules in gene interaction network was used to find transcripts which are altered in abundance in AML samples when compared to normal samples. This algorithm focused on finding small networks, which would be easier to be interpreted and validated. It computes *P* values for subnetworks, which helped identify significant subnetworks. This analysis produced lists of significant sub-networks (Supplemental Table  2). 

### 2.8. Final Protein Target Selection

 The objective of the protein target selection was to analyze data generated through chemoinformatics and structural informatics method, and select relevant and important predicted binding proteins, which would be used for selecting potential drug/drug combination. Detailed literature searches were conducted for predicted binding proteins generated by the earlier analyses (Target Discovery/Identification). The disease pathways annotations from KEGG were used to select cancer related predicted binding proteins along with additional literature searches. The literature searches were used to support annotations from KEGG and also to provide additional information about target protein whose role in carcinogenesis has been established recently [13–24].

*Predicted binding proteins were selected based on following criteria*:

1. Target protein should be predicted as related with compounds of interest (natural products estimated from Reverse Docking or Ontomine).
2. Predicted binding proteins should be related with AML/cancer (KEGG annotation and/or literature).

### 2.9. Validation of Existing Drugs for AML

 Seventeen existing drugs for AML were included in the analysis to (i) understand the mode of action of drugs; (ii) predict activity profiles; and (iii) use them as benchmarks for the natural products analysis. The following drugs were used as benchmark sets in analysis; natural products 6-mercaptopurine; 6-thioguanine; L-malate and vincristine; and synthesized drugs, amonafide, belinostat, clofarabine, cytarabine, daunorubicin, etoposide, fludarabine, gemcitabine, idarubicin, mitoxantrone, paclitaxel, prednisone, and tipifarnib. Reverse docking for 13 natural products along with 17 benchmark drugs was carried out with a target database (PDTD) of ~1060 protein structures. Each Docking was performed using 50,000 energy evaluations for 5 conformational searches per ligand, with a 60 × 60 × 60 dimensional grid box size and a 0.375 Å grid covering the whole of the active site. The predicted binding proteins were further screened using the cut off value of 10 *μ*M for the inhibition constant (*K*_*i*_).

### 2.10. Drug Combination Selection

 Searching potential interactions among 13 natural products required a factorial design to consider all possible combinations (78 pairs or 286 sets of 3 natural products). The search algorithm was designed to find potential nodes in the tree structure where root nodes represented singlet drugs and processes. Finding interactions involved moving up the tree to explore combinations. This approach helped to avoid false positives by limiting the search space to significant nodes and processes. Combiscores (hereafter Cscores) were used in drug/drug-combination ranking and selection. The Cscore was derived by analyzing target profile by Ontomine for drug combinations where

(2) Cscore=(0.2∗(Ac  −  (Am+Ah))+(0.4  ∗Am)  +(0.3∗Ah))−  0.1∗comm+sp,

where *Ac* was the number of predicted binding proteins related to cancer; *Am* was the number of predicted binding proteins related to AML; *Ah* was the number of predicted binding proteins detected as hubs in interaction networks from gene-expression network analysis; comm was the number of common target protein(s) shared by constituent drugs in combination; sp was the specificity score defined as (the number of cancer-related predicted binding proteins minus the number of protein not related to cancer)/Total no. of predicted binding proteins for drug/combination).

 Dscore was derived by analyzing target profile obtained through docking analysis for drug combinations where

(3) Dscore=((0.2∗(Ac−(Am+Ah))+  (0.4∗Am)) +(0.3∗Ah))−0.1∗comm)N−1;

*N* was the number of proteins used for forward docking (*N* = 59).

Combiscore: was the statistic derived from combining Cscore and Dscore:

(4) $$\begin{array}{c} \text{Combiscore}\;=\;\left(0.6\times \text{Cscore}\right)\;+\;\left(0.4\;\times \;\text{Dscore}\right). \end{array}$$

Computing the scores weights was associated with parameters, to alter their relative importance. For example predicted binding proteins related with AML were given more weight than proteins associated with cancers in general. For single natural products the process began by computing the Combiscore for each of the 13 natural products. The mean Combiscore was calculated (1.36) and natural products with Combiscore greater or equal to 1.36 were selected as potentially useful. 

 For pairs of natural products all possible binary combinations were considered, with constraint of considering only those combinations which started from a selected singlet. A Combiscore was computed for each pair and ranked. The mean Combiscore was calculated. Finally, potential combinations that had a Combiscore greater or equal to the mean Combiscore (4.25) were reported. 

 For sets of three natural products combinations the same process was followed with the Combiscore threshold set greater or equal to the mean Combiscore (6.15). Higher Combiscores indicated better drug combinations.

## 3. Results

### 3.1. Bioassays

Ontomine analysis identified probable targets for each of the 13 natural products among proteins that were potential bioassay targets (Table 2). In total 618 proteins were predicted to be bioassay targets at various confidence levels (157 at high confidence, 91 at medium confidence and 370 at low confidence). Table 1 showed the four proteins previously reported to be bioassays targets of the natural products. Multiple (4–8) natural products were predicted to interact with bioassays targets, guanine nucleotide binding protein, alpha activating polypeptide O, multidrug resistance protein 1, myeloid or lymphoid or mixed-lineage leukaemia protein, and runt-related transcription factor isoform 1 (AML1c). Clearly Ontomine identified a larger set of bioassays than what has been previously reported. Those bioassay targets could be contributing to the effects or side-effects of the natural products.

### 3.2. Reverse Docking

 Reverse docking was predicted with structures of natural products docked against protein databases. Using the structures of the natural product to interrogate the database of protein structures identified 17 proteins predicted to bind natural products with a *K*_*i*_ less than 1 *μ*M. All 17 were predicted to be related to AML by network analysis. They were ALOX12 that encoded arachidonate 12-lipoxygenase; AR for the androgen receptor; BCL2L1 and BCL2L2 for the B-cell lymphoma 2-like proteins; CDC42 encoding the cell division cycle 42 (GTP binding protein, 25 kDa); DUSP3 for the dual specificity phosphatase 3; GSK3B encoded the glycogen synthase kinase 3 beta; IGF1R for the insulin-like growth factor 1 receptor; KLF5 for the ruppel-like factor 5 (intestinal); MAPK1 for the mitogen-activated protein kinase 1; MMP14 for the matrix metallopeptidase 14 (membrane inserted) protein; NFKB1 that encoded the nuclear factor of kappa light polypeptide gene enhancer in B cells; RHOA, the ras homolog gene family, member A; RUNX1, the runt-related transcription factor 1; SMAD3, for mothers against decapentaplegic homolog family member 3; and STAT1 and STAT3 the signal transducer and activator of transcription 91 kDa protein and acute-phase response factor, respectively. In Figure 1 three of these proteins (STAT3, MAPK1, IGF1R) were found within a small node of just fifteen interacting proteins. Further, two proteins were involved in B-cell regulation (BCL-2, KFKB1).

In total reverse docking for 13 natural products and 17 drugs to ~1,060 protein structures yielded 92 targets with inhibition constants (*K*_*i*_) < 10 *μ*M. The reverse docking results were then analysed in conjunction with the Ontomine results. These include targets which either belong to a subset of reverse docking and study or else they are identified as important targets by Ontomine. A total of 95 targets were finally identified (Supplemental Table 3). 

### 3.3. Docking

Among the 95 binding proteins identified by Reverse Docking, the protein structures which could be used for docking were available for 59 targets. These 59 structures were finally used for docking against the 14 ligand structures under consideration along with the benchmark ligands. The docking parameters were kept the same as those used for reverse docking. 

**Figure 2:**
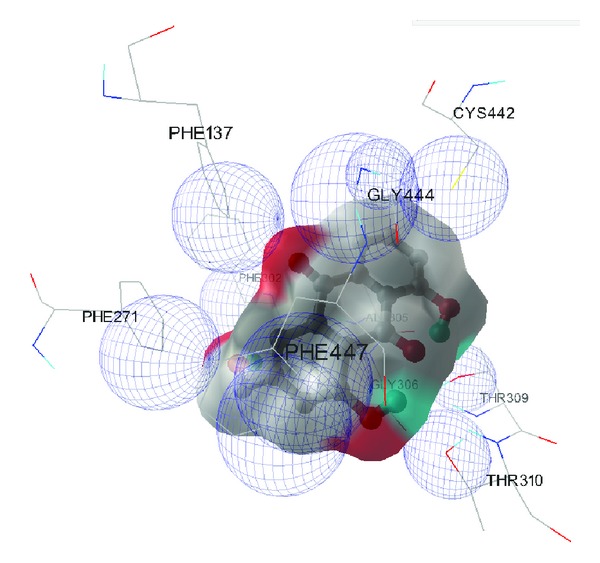
Aloe emodin was predicted to be bound at the centre surrounded by the amino acid residues from the active site of 2GDZ. It can be seen that aloe emodin forms 4 hydrogen bonded interactions with the residues of the active site. These hydrogen bonded interactions are partly responsible for stabilizing the ligand-protein complex. In addition the hydrophobic interactions present between the ligand and the protein are displayed as wireframe spheres.

Among the ligand-protein binding interactions of significance was aloe emodin predicted to bind to the protein 15-hydroxyprostaglandin dehydrogenase type1 (2GDZ; Figure 2). Aloe emodin was predicted to bind at the center of the structure surrounded by the amino acid residues from the active site of 2GDZ. It can be seen that aloe emodin forms 4 hydrogen bond interactions with the residues of the active site. These hydrogen bond interactions were predicted to stabilize the ligand-protein complex. The human 15-hydroxyprostaglandin dehydrogenase type1 has been reported to be elevated in abundance and activity in AML cell lines [7].

### 3.4. Combinations of Natural Products and Drugs for AML Treatments

The Cscore,Dscore and Combiscores were all significant for aloe emodin, chrysin, honokiol, mevinolin, resveratrol, l-ascorbic acid, 6- palmitate, and cholecalciferol (Table 3 (a)). Considering pairs of natural products with predicted synergistic interactions, 25 combinations were found (Table 3 (b)). Twelve pairs included aloe emodin suggesting that this natural product would work well in many mixtures. There were 7 pairs that included honokiol and 6 for chrysin. Even natural products not predicted to be effective alone, like limonene, could be effective in paired mixtures.

**Figure 3:**
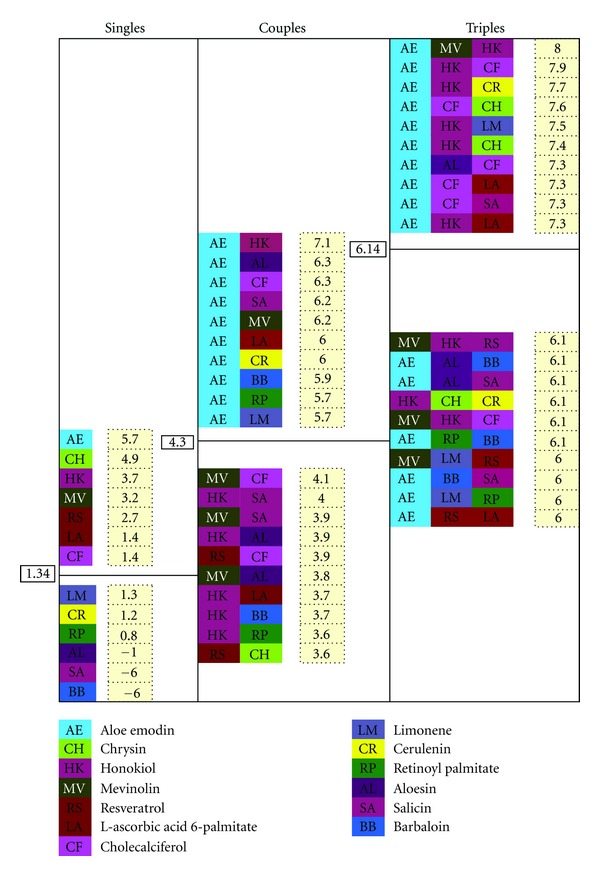
Drug combination selections. Values inside beige squares were the Combiscores. Each black box was the mean used to derive the cutoff.

Considering sets of three natural products with predicted synergistic interactions 15 combinations were found (Table 3 (b)). Eleven drug combinations included aloe emodin, again suggesting that this natural product would work well in many mixtures. There were 5 mixtures that included honokiol but only 3 for chrysin. Again natural product not predicted to be effective alone like limonene could be effective in multiple mixtures (Figure 3).

## 4. Discussion

Analysis of 13 natural products inferred that some interactions might be useful and novel. Target profiling by Ontomine, Docking, and Gene Expression Network analysis indicated that the natural products were predicted to bind to proteins which were important targets for cancer treatments. Pathway analyses indicated statistical overrepresentation of cancer-related pathways among drug targets for aloe emodin, cerulenin, chrysin, honokiol, mevinolin, and resveratrol. 

 Mixtures of natural products were predicted to be more effective than single products, as reported experimentally [7, 8, 26]. Improvements in predicted effectiveness for mixtures of natural products could be attributed to drug synergism due to increase in relevant targets or improved specificity of drug constituents. This increase in predicted effectiveness was not likely to be derived from random effects like pooling of result of individual drugs, since the analysis accounted for important factors for drug combinations like target relevance to cancer/AML, specificity, and common targets among drug constituents, while calculating scores are used for ranking drug combination.

The parallel analysis on benchmarked drugs which exist in the market for AML treatment was significant since the efficacy of these drugs was supported by many publications [2, 8, 10, 27]. Combination analysis on drugs was also successful in discovering well-known combinations like amonafide plus cytarabine and daunorubicin plus prednisone. That aloe emodin plus mevinolin plus honokiol was identified as the best combination that has interesting clinical implications. Analysis of this mixture with respect to drug specificity, targeting AML-related proteins and targeting cancer-related hubs, will be a priority for future laboratory and clinical research. Several other combinations have potential to treat drug-resistant cancers in the future. In future analyses *in silico* more complex drug combination search algorithms can be applied to incorporate dose, absorption, and excretion as suggested [27].

## Supplementary Material

Supplementary materials aims to provide more details of drug target database which was used for analysis as well the toxicities predicted for natural products, list of targets identified by Ontomine and list of cancer related proteins used for forward docking to natural products.

## Figures and Tables

**Table 1:** Bioassay targets are confirmed as AML active proteins by literature searches and associated binding proteins are predicted by Ontomine.

S. no	Ontomine predicted bioassays	Confidence level	Compound(s)	Reference no
1	Guanine nucleotide binding protein, alpha activating polypeptide-O	Medium	Honokiol	[13]
		Low	Cholecalciferol Limonene Retinyl palmitate	

2	Multidrug resistance protein 1	Low	Mevinolin Limonene Resveratrol Chrysin	[14]

3	Myeloid or lymphoid or mixed-lineage leukaemia protein	High	Aloe emodin Mevinolin	[15]
		Low	Barbaloin Limonene Retinyl palmitate Cholecalciferol Chrysin L-Ascorbic acid 6-palmitate	

4	Runt-related transcription factor 1 isoform AML1c	High	Chrysin	[16]
		Medium	Honokiol	
		Low	Barbaloin Mevinolin Limonene L-Ascorbic acid 6-palmitate	

**Table 2:** Protein targets for natural products previously reported in the literature.

S. no	Ontomine predicted compound(s)	Bioassay targets	References no
1	Mevinolin	Multidrug resistance protein 1	[17]
2	Limonene	Multidrug resistance protein 1	[18]
3	Resveratrol	Multidrug resistance protein 1	[19]
4	Chrysin	Multidrug resistance protein 1	[20]
5	Cholecalciferol	Myeloid or lymphoid or mixed-lineage leukaemia protein	[21]

**Table:** (a) Singlet analysis

Compound	Cscore	Dscore	Combiscore
Aloe emodin	**9.409090909**	**0.027118644**	**5.656302003**
Chrysin	**8.2**	**0.044067797**	**4.937627119**
Honokiol	**6.133333333**	**0.054237288**	**3.701694915**
Mevinolin	**5.35**	**0.020338983**	**3.218135593**
Resveratrol	**4.559259259**	**0.013559322**	**2.740979284**
L-ascorbic acid 6-palmitate	**2.376470588**	**0**	**1.425882353**
Cholecalciferol	**2.25**	**0.025423729**	**1.360169492**
Limonene	2.230769231	0.018644068	1.345919166
Cerulenin	2	0.038983051	1.21559322
Retinyl palmitate	1.4	0.005084746	0.842033898
Aloe sin	−1	0.025423729	−0.589830508
Salicin	−10	0.052542373	−5.978983051
Barbaloin	−10	0.037288136	−5.985084746

**Table:** (b) List of binary combinations selected by analysis

Compound	Cscore	Dscore	Combiscore
Aloe emodin + honokiol	**10.33333333**	**2.125423729**	**7.050169492**
Aloe emodin + Aloe sin	**8.833333333**	**2.588135593**	**6.335254237**
Aloe emodin + cholecalciferol	**10.25384615**	**0.376271186**	**6.302816167**
Aloe emodin + salicin	**8.520754717**	**2.806779661**	**6.235164695**
Aloe emodin + mevinolin	**9.523076923**	**1.154237288**	**6.175541069**
Aloe emodin + l-ascorbic acid 6-palmitate	**8.968421053**	**1.637288136**	**6.035967886**
Aloe emodin + cerulenin	**9.561538462**	**0.610169492**	**5.980990874**
Aloe emodin + barbaloin	**8.520754717**	**1.93220339**	**5.885334186**
Aloe emodin + retinylpalmitate	**8.945454545**	**0.86440678**	**5.713035439**
Aloe emodin + limonene	**9.255932203**	**0.376271186**	**5.704067797**
Aloe emodin + resveratrol	**7.910344828**	**2.313559322**	**5.671630625**
Chrysin + l-ascorbic acid 6-palmitate	**8.711764706**	**0.503389831**	**5.428414756**
Chrysin + cerulenin	**8.784615385**	**0.284745763**	**5.384667536**
Chrysin + salicin	**7.838461538**	**1.672881356**	**5.372229465**
Aloe emodin + chrysin	**7.87704918**	**1.418644068**	**5.293687135**
Chrysin + barbaloin	**7.838461538**	**0.798305085**	**5.022398957**
Mevinolin + chrysin	**7.77704918**	**0.569491525**	**4.894026118**
Cholecalciferol + chrysin	**6.881481481**	**1.698305085**	**4.808210923**
Honokiol + resveratrol	**7.696296296**	**0.354237288**	**4.759472693**
Honokiol + limonene	**7.775**	**0.166101695**	**4.731440678**
Mevinolin + resveratrol	**6.824489796**	**1.46440678**	**4.680456589**
Honokiol + chrysin	**7.609836066**	**0.166101695**	**4.632342317**
Honokiol + cholecalciferol	**7.433333333**	**0.166101695**	**4.526440678**
Mevinolin + honokiol	**6.385714286**	**1.276271186**	**4.341937046**
Honokiol + cerulenin	**7.030769231**	**0.166101695**	**4.284902216**
Mevinolin + limonene	**6.885714286**	**0.305084746**	**4.25346247**
Mevinolin + cholecalciferol	6.572727273	0.305084746	4.065670262
Honokiol + salicin	6.033333333	0.847457627	3.958983051
Mevinolin + salicin	5.15	1.957627119	3.873050847
…	…	…	…

**Table:** (c) List of triple combinations selected by analysis

Compound	Cscore	Dscore	Combiscore
Aloe emodin + mevinolin + honokiol	**12.00864198**	**1.866101695**	**7.951625863**
Aloe emodin + honokiol + cholecalciferol	**11.74146341**	**2.125423729**	**7.89504754**
Aloe emodin + honokiol + cerulenin	**11.49268293**	**2.047457627**	**7.714592807**
Aloe emodin + cholecalciferol + chrysin	**11.78888889**	**1.418644068**	**7.64079096**
Aloe emodin + honokiol + limonene	**11.15064935**	**2.125423729**	**7.540559102**
Aloe emodin + honokiol + chrysin	**11.15064935**	**1.777966102**	**7.401576051**
Aloe emodin + Aloe sin + cholecalciferol	**10.45384615**	**2.588135593**	**7.30756193**
…	**…**	**…**	**…**
Mevinolin + chrysin + barbaloin	**9.87704918**	**0.994915254**	**6.32419561**
Aloe emodin + resveratrol + cerulenin	**9.046376812**	**2.23559322**	**6.322063375**
Mevinolin + chrysin + l-ascorbic acid 6-palmitate	**10.06507937**	**0.7**	**6.319047619**
Mevinolin + chrysin + cerulenin	**10.11343284**	**0.569491525**	**6.295856312**
Aloe emodin + mevinolin + l-ascorbic acid 6-palmitate	**9.523076923**	**1.377966102**	**6.265032595**
Aloe emodin + mevinolin + retinylpalmitate	**9.733333333**	**0.991525424**	**6.236610169**
Mevinolin + honokiol + limonene	**9.433333333**	**1.276271186**	**6.170508475**
Honokiol + limonene + resveratrol	**10.02258065**	**0.354237288**	**6.155243302**
Mevinolin + honokiol + resveratrol	9.464705882	1.140677966	6.135094716
Aloe emodin + Aloe sin + barbaloin	8.833333333	2.069491525	6.12779661
…	…	…	…
