# Supplementary material for: Predicting In Silico Which Mixtures of the Natural Products of Plants Might Most Effectively Kill Human Leukemia Cells?
Source: Evid Based Complement Alternat Med. 2013 Jan 28;2013:801501. doi: 10.1155/2013/801501 (PMC3569894; doi:10.1155/2013/801501)
Supplement: Supplementary file 1 — Supplementary materials aims to provide more details of drug target database which was used for analysis as well the toxicities predicted for natural products, list of targets identified by Ontomine and list of cancer related proteins used for forward docking to natural products. [file 801501.f1.doc]

### Supplementary Analysis

In addition to drug combination analysis, we have also performed toxicity and other physiochemical properties analysis for 13 natural compounds by Ontomine.

Supplemental Table 1:Panel A: Toxicities predicted for natural compounds

### ADME-Physicochemical Properties Profiling

Supplemental Table 1: Panel B; Aqueous Solubility predictions

Supplemental Table 1 : Panel C: Partition coefficents (logP) prediction results

*Supplemental Table 2*

1. Table2: List of targets identified by Ontomine^TM^ Reverse Docking tool, which were also predicted by the Network Analysis tool.

*Supplemental Table 3*
